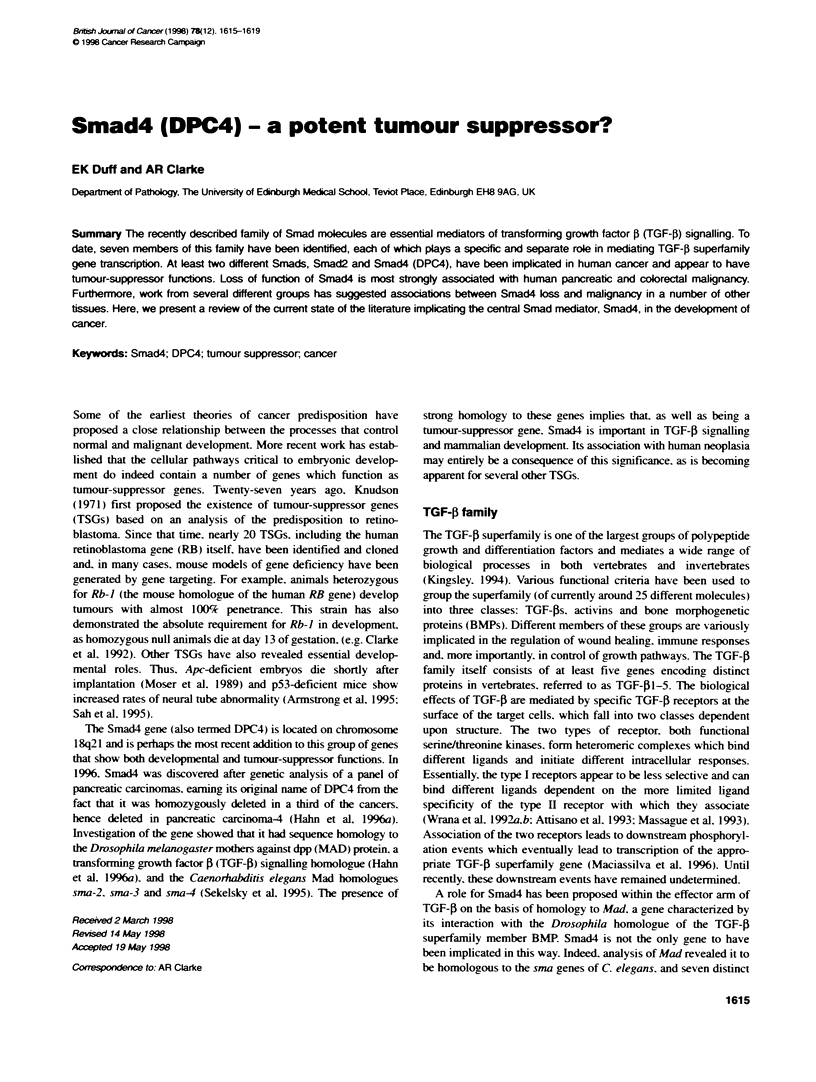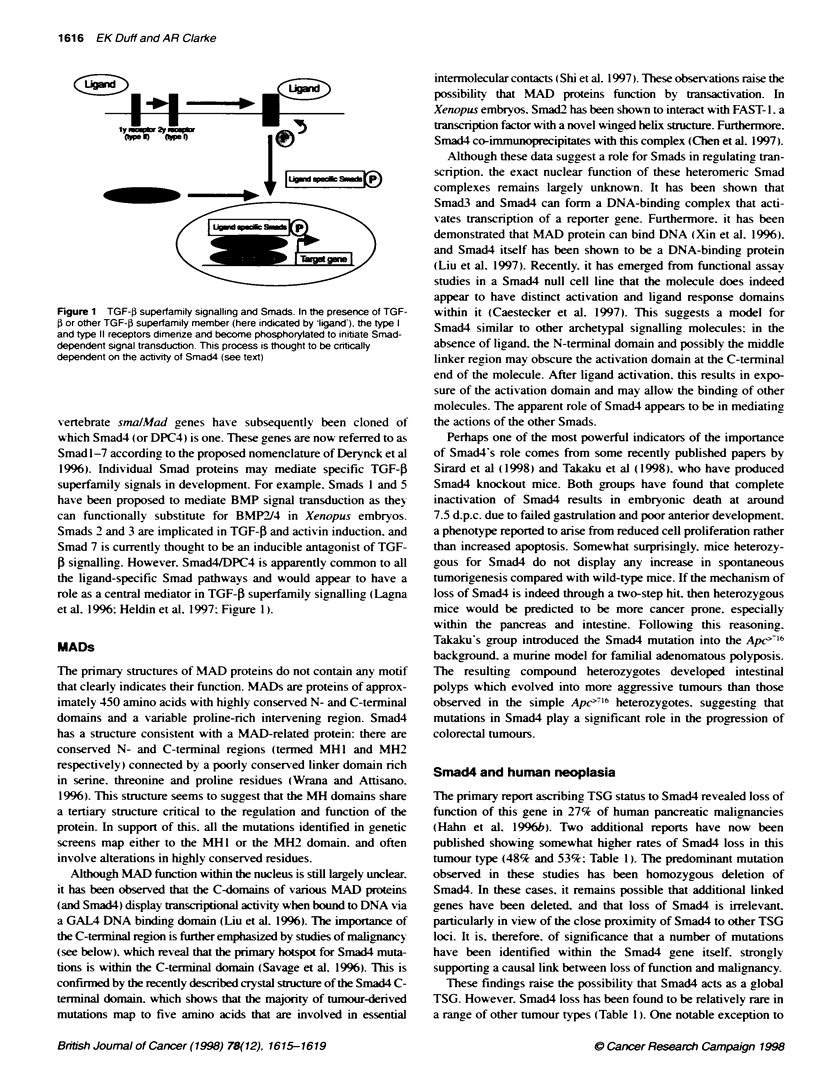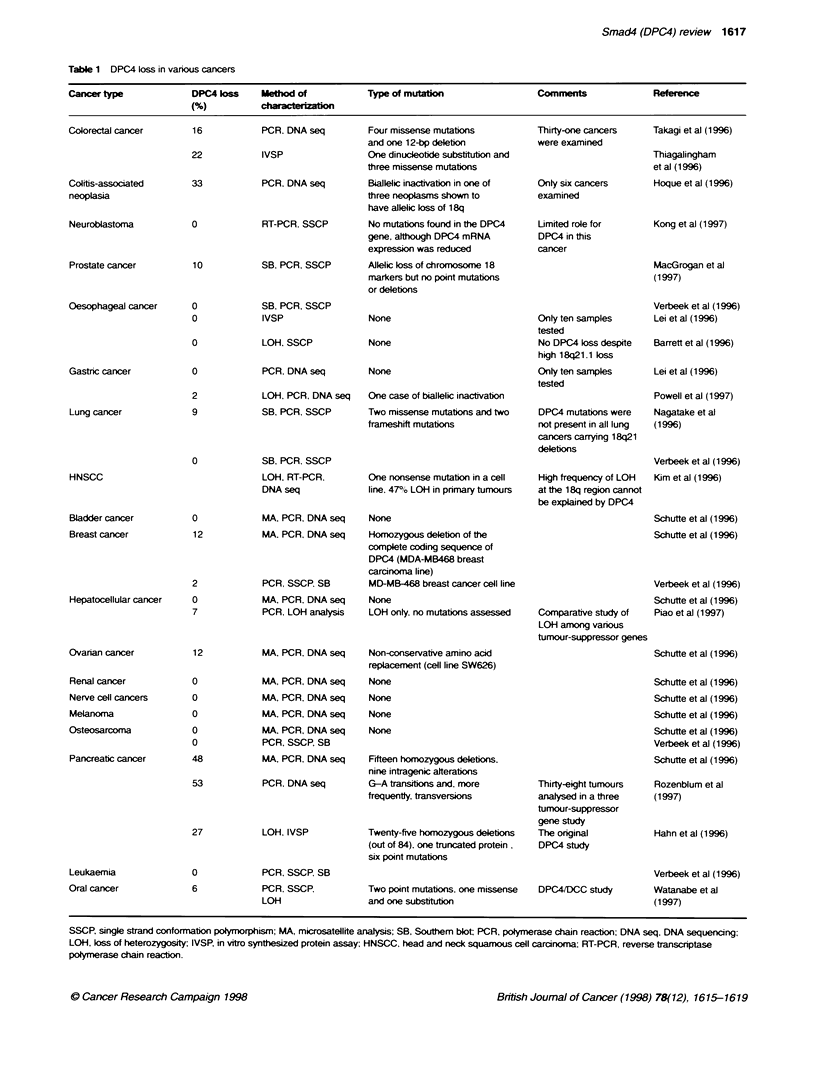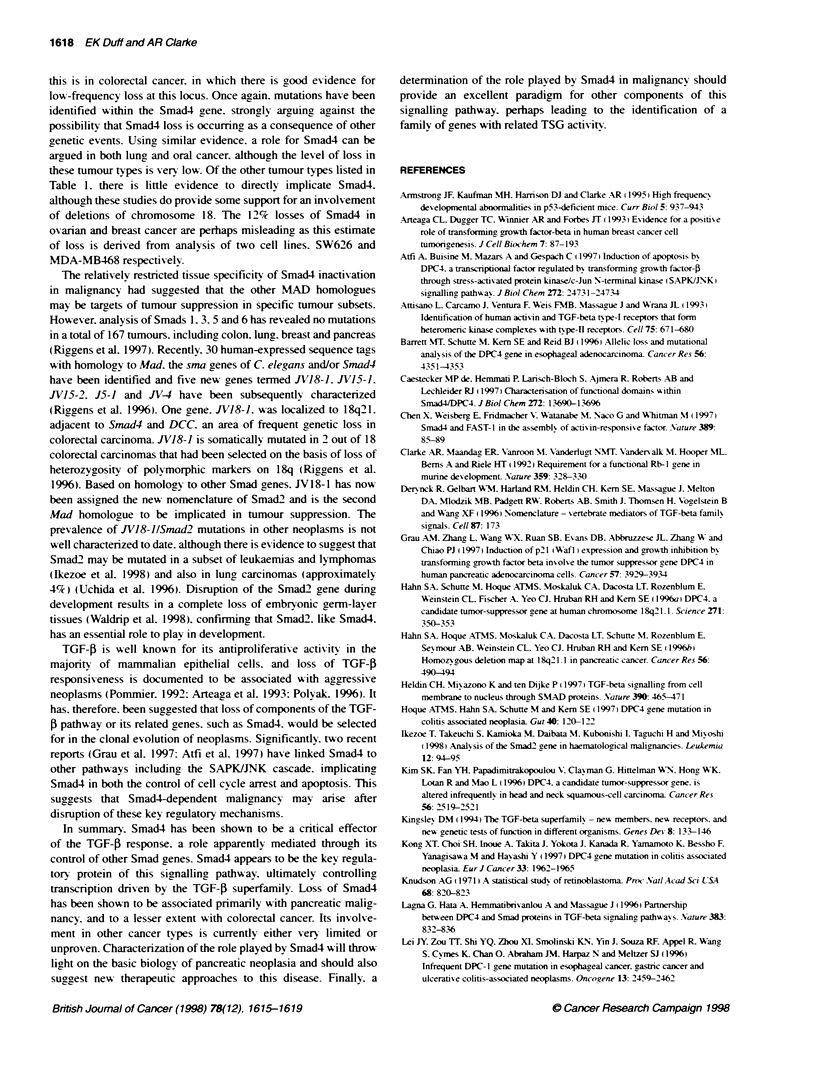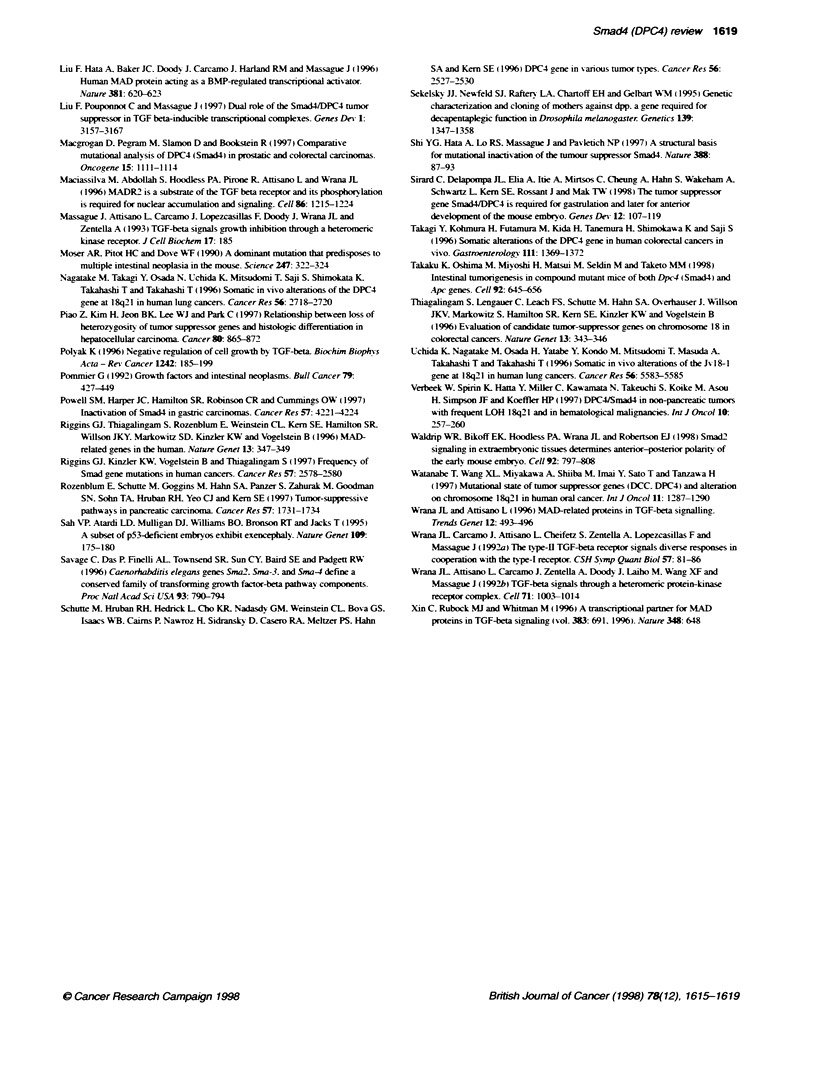# Smad4 (DPC4)--a potent tumour suppressor?

**DOI:** 10.1038/bjc.1998.731

**Published:** 1998-12

**Authors:** E. K. Duff, A. R. Clarke

**Affiliations:** Department of Pathology, The University of Edinburgh Medical School, UK.

## Abstract

The recently described family of Smad molecules are essential mediators of transforming growth factor beta (TGF-beta) signalling. To date, seven members of this family have been identified, each of which plays a specific and separate role in mediating TGF-beta superfamily gene transcription. At least two different Smads, Smad2 and Smad4 (DPC4), have been implicated in human cancer and appear to have tumour-suppressor functions. Loss of function of Smad4 is most strongly associated with human pancreatic and colorectal malignancy. Furthermore, work from several different groups has suggested associations between Smad4 loss and malignancy in a number of other tissues. Here, we present a review of the current state of the literature implicating the central Smad mediator, Smad4, in the development of cancer.


British Journal of Cancer (1998) 78(12). 1615–1619
© 1998 Cancer Research Campaign

### Smad4 (DPC4) – a potent tumour suppressor?

**EK Duff and AR Clarke**

Department of Pathology, The University of Edinburgh Medical School, Teviot Place, Edinburgh EH8 9AG, UK

**Summary** The recently described family of Smad molecules are essential mediators of transforming growth factor β (TGF-β) signalling. To date, seven members of this family have been identified, each of which plays a specific and separate role in mediating TGF-β superfamily gene transcription. At least two different Smads, Smad2 and Smad4 (DPC4), have been implicated in human cancer and appear to have tumour-suppressor functions. Loss of function of Smad4 is most strongly associated with human pancreatic and colorectal malignancy. Furthermore, work from several different groups has suggested associations between Smad4 loss and malignancy in a number of other tissues. Here, we present a review of the current state of the literature implicating the central Smad mediator, Smad4, in the development of cancer.

**Keywords:** Smad4; DPC4; tumour suppressor; cancer

Some of the earliest theories of cancer predisposition have proposed a close relationship between the processes that control normal and malignant development. More recent work has established that the cellular pathways critical to embryonic development do indeed contain a number of genes which function as tumour-suppressor genes. Twenty-seven years ago, Knudson (1971) first proposed the existence of tumour-suppressor genes (TSGs) based on an analysis of the predisposition to retinoblastoma. Since that time, nearly 20 TSGs, including the human retinoblastoma gene (RB) itself, have been identified and cloned and, in many cases, mouse models of gene deficiency have been generated by gene targeting. For example, animals heterozygous for *Rb-1* (the mouse homologue of the human *RB* gene) develop tumours with almost 100% penetrance. This strain has also demonstrated the absolute requirement for *Rb-1* in development, as homozygous null animals die at day 13 of gestation, (e.g. Clarke et al, 1992). Other TSGs have also revealed essential developmental roles. Thus, *Apc*-deficient embryos die shortly after implantation (Moser et al, 1989) and p53-deficient mice show increased rates of neural tube abnormality (Armstrong et al, 1995; Sah et al, 1995).

The Smad4 gene (also termed DPC4) is located on chromosome 18q21 and is perhaps the most recent addition to this group of genes that show both developmental and tumour-suppressor functions. In 1996, Smad4 was discovered after genetic analysis of a panel of pancreatic carcinomas, earning its original name of DPC4 from the fact that it was homozygously deleted in a third of the cancers, hence deleted in pancreatic carcinoma-4 (Hahn et al, 1996*a*). Investigation of the gene showed that it had sequence homology to the *Drosophila melanogaster* mothers against dpp (MAD) protein, a transforming growth factor β (TGF-β) signalling homologue (Hahn et al, 1996*a*), and the *Caenorhabditis elegans* Mad homologues *sma-2*, *sma-3* and *sma-4* (Sekelsky et al, 1995). The presence of strong homology to these genes implies that, as well as being a tumour-suppressor gene, Smad4 is important in TGF-β signalling and mammalian development. Its association with human neoplasia may entirely be a consequence of this significance, as is becoming apparent for several other TSGs.

### TGF-β family

The TGF-β superfamily is one of the largest groups of polypeptide growth and differentiation factors and mediates a wide range of biological processes in both vertebrates and invertebrates (Kingsley, 1994). Various functional criteria have been used to group the superfamily (of currently around 25 different molecules) into three classes: TGF-βs, activins and bone morphogenetic proteins (BMPs). Different members of these groups are variously implicated in the regulation of wound healing, immune responses and, more importantly, in control of growth pathways. The TGF-β family itself consists of at least five genes encoding distinct proteins in vertebrates, referred to as TGF-β1–5. The biological effects of TGF-β are mediated by specific TGF-β receptors at the surface of the target cells, which fall into two classes dependent upon structure. The two types of receptor, both functional serine/threonine kinases, form heteromeric complexes which bind different ligands and initiate different intracellular responses. Essentially, the type I receptors appear to be less selective and can bind different ligands dependent on the more limited ligand specificity of the type II receptor with which they associate (Wrana et al, 1992*a*,*b*; Attisano et al, 1993; Massague et al, 1993). Association of the two receptors leads to downstream phosphorylation events which eventually lead to transcription of the appropriate TGF-β superfamily gene (Maciassilva et al, 1996). Until recently, these downstream events have remained undetermined.

A role for Smad4 has been proposed within the effector arm of TGF-β on the basis of homology to *Mad*, a gene characterized by its interaction with the *Drosophila* homologue of the TGF-β superfamily member BMP. Smad4 is not the only gene to have been implicated in this way. Indeed, analysis of *Mad* revealed it to be homologous to the *sma* genes of *C. elegans*, and seven distinct

*Received 2 March 1998*
*Revised 14 May 1998*
*Accepted 19 May 1998*

*Correspondence to:* AR Clarke

**Figure 1** TGF-β superfamily signalling and Smads. In the presence of TGF-β or other TGF-β superfamily member (here indicated by 'ligand'), the type I and type II receptors dimerize and become phosphorylated to initiate Smad-dependent signal transduction. This process is thought to be critically dependent on the activity of Smad4 (see text)

vertebrate *sma/Mad* genes have subsequently been cloned of which Smad4 (or DPC4) is one. These genes are now referred to as Smad1–7 according to the proposed nomenclature of Derynck et al 1996). Individual Smad proteins may mediate specific TGF-β superfamily signals in development. For example, Smads 1 and 5 have been proposed to mediate BMP signal transduction as they can functionally substitute for BMP2/4 in *Xenopus* embryos. Smads 2 and 3 are implicated in TGF-β and activin induction, and Smad 7 is currently thought to be an inducible antagonist of TGF-β signalling. However, Smad4/DPC4 is apparently common to all the ligand-specific Smad pathways and would appear to have a role as a central mediator in TGF-β superfamily signalling (Lagna et al, 1996; Heldin et al, 1997; Figure 1).

### MADs

The primary structures of MAD proteins do not contain any motif that clearly indicates their function. MADs are proteins of approximately 450 amino acids with highly conserved N- and C-terminal domains and a variable proline-rich intervening region. Smad4 has a structure consistent with a MAD-related protein: there are conserved N- and C-terminal regions (termed MH1 and MH2 respectively) connected by a poorly conserved linker domain rich in serine, threonine and proline residues (Wrana and Attisano, 1996). This structure seems to suggest that the MH domains share a tertiary structure critical to the regulation and function of the protein. In support of this, all the mutations identified in genetic screens map either to the MH1 or the MH2 domain, and often involve alterations in highly conserved residues.

Although MAD function within the nucleus is still largely unclear, it has been observed that the C-domains of various MAD proteins (and Smad4) display transcriptional activity when bound to DNA via a GAL4 DNA binding domain (Liu et al, 1996). The importance of the C-terminal region is further emphasized by studies of malignancy (see below), which reveal that the primary hotspot for Smad4 mutations is within the C-terminal domain (Savage et al, 1996). This is confirmed by the recently described crystal structure of the Smad4 C-terminal domain, which shows that the majority of tumour-derived mutations map to five amino acids that are involved in essential intermolecular contacts (Shi et al, 1997). These observations raise the possibility that MAD proteins function by transactivation. In *Xenopus* embryos, Smad2 has been shown to interact with FAST-1, a transcription factor with a novel winged helix structure. Furthermore, Smad4 co-immunoprecipitates with this complex (Chen et al, 1997).

Although these data suggest a role for Smads in regulating transcription, the exact nuclear function of these heteromeric Smad complexes remains largely unknown. It has been shown that Smad3 and Smad4 can form a DNA-binding complex that activates transcription of a reporter gene. Furthermore, it has been demonstrated that MAD protein can bind DNA (Xin et al, 1996), and Smad4 itself has been shown to be a DNA-binding protein (Liu et al, 1997). Recently, it has emerged from functional assay studies in a Smad4 null cell line that the molecule does indeed appear to have distinct activation and ligand response domains within it (Caestecker et al, 1997). This suggests a model for Smad4 similar to other archetypal signalling molecules: in the absence of ligand, the N-terminal domain and possibly the middle linker region may obscure the activation domain at the C-terminal end of the molecule. After ligand activation, this results in exposure of the activation domain and may allow the binding of other molecules. The apparent role of Smad4 appears to be in mediating the actions of the other Smads.

Perhaps one of the most powerful indicators of the importance of Smad4's role comes from some recently published papers by Sirard et al (1998) and Takaku et al (1998), who have produced Smad4 knockout mice. Both groups have found that complete inactivation of Smad4 results in embryonic death at around 7.5 d.p.c. due to failed gastrulation and poor anterior development, a phenotype reported to arise from reduced cell proliferation rather than increased apoptosis. Somewhat surprisingly, mice heterozygous for Smad4 do not display any increase in spontaneous tumorigenesis compared with wild-type mice. If the mechanism of loss of Smad4 is indeed through a two-step hit, then heterozygous mice would be predicted to be more cancer prone, especially within the pancreas and intestine. Following this reasoning, Takaku's group introduced the Smad4 mutation into the *Apc*$^{\Delta 716}$ background, a murine model for familial adenomatous polyposis. The resulting compound heterozygotes developed intestinal polyps which evolved into more aggressive tumours than those observed in the simple *Apc*$^{\Delta 716}$ heterozygotes, suggesting that mutations in Smad4 play a significant role in the progression of colorectal tumours.

### Smad4 and human neoplasia

The primary report ascribing TSG status to Smad4 revealed loss of function of this gene in 27% of human pancreatic malignancies (Hahn et al, 1996*b*). Two additional reports have now been published showing somewhat higher rates of Smad4 loss in this tumour type (48% and 53%; Table 1). The predominant mutation observed in these studies has been homozygous deletion of Smad4. In these cases, it remains possible that additional linked genes have been deleted, and that loss of Smad4 is irrelevant, particularly in view of the close proximity of Smad4 to other TSG loci. It is, therefore, of significance that a number of mutations have been identified within the Smad4 gene itself, strongly supporting a causal link between loss of function and malignancy.

These findings raise the possibility that Smad4 acts as a global TSG. However, Smad4 loss has been found to be relatively rare in a range of other tumour types (Table 1). One notable exception to

 © Cancer Research Campaign 1998

**Table 1** DPC4 loss in various cancers

| Cancer type | DPC4 loss (%) | Method of characterization | Type of mutation | Comments | Reference |
|---|---|---|---|---|---|
| Colorectal cancer | 16 | PCR, DNA seq | Four missense mutations and one 12-bp deletion | Thirty-one cancers were examined | Takagi et al (1996) |
| | 22 | IVSP | One dinucleotide substitution and three missense mutations | | Thiagalingham et al (1996) |
| Colitis-associated neoplasia | 33 | PCR, DNA seq | Biallelic inactivation in one of three neoplasms shown to have allelic loss of 18q | Only six cancers examined | Hoque et al (1996) |
| Neuroblastoma | 0 | RT-PCR, SSCP | No mutations found in the DPC4 gene, although DPC4 mRNA expression was reduced | Limited role for DPC4 in this cancer | Kong et al (1997) |
| Prostate cancer | 10 | SB, PCR, SSCP | Allelic loss of chromosome 18 markers but no point mutations or deletions | | MacGrogan et al (1997) |
| Oesophageal cancer | 0 | SB, PCR, SSCP | | | Verbeek et al (1996) |
| | 0 | IVSP | None | Only ten samples tested | Lei et al (1996) |
| | 0 | LOH, SSCP | None | No DPC4 loss despite high 18q21.1 loss | Barrett et al (1996) |
| Gastric cancer | 0 | PCR, DNA seq | None | Only ten samples tested | Lei et al (1996) |
| | 2 | LOH, PCR, DNA seq | One case of biallelic inactivation | | Powell et al (1997) |
| Lung cancer | 9 | SB, PCR, SSCP | Two missense mutations and two frameshift mutations | DPC4 mutations were not present in all lung cancers carrying 18q21 deletions | Nagatake et al (1996) |
| | 0 | SB, PCR, SSCP | | | Verbeek et al (1996) |
| HNSCC | | LOH, RT-PCR, DNA seq | One nonsense mutation in a cell line, 47% LOH in primary tumours | High frequency of LOH at the 18q region cannot be explained by DPC4 | Kim et al (1996) |
| Bladder cancer | 0 | MA, PCR, DNA seq | None | | Schutte et al (1996) |
| Breast cancer | 12 | MA, PCR, DNA seq | Homozygous deletion of the complete coding sequence of DPC4 (MDA-MB468 breast carcinoma line) | | Schutte et al (1996) |
| | 2 | PCR, SSCP, SB | MD-MB-468 breast cancer cell line | | Verbeek et al (1996) |
| Hepatocellular cancer | 0 | MA, PCR, DNA seq | None | | Schutte et al (1996) |
| | 7 | PCR, LOH analysis | LOH only, no mutations assessed | Comparative study of LOH among various tumour-suppressor genes | Piao et al (1997) |
| Ovarian cancer | 12 | MA, PCR, DNA seq | Non-conservative amino acid replacement (cell line SW626) | | Schutte et al (1996) |
| Renal cancer | 0 | MA, PCR, DNA seq | None | | Schutte et al (1996) |
| Nerve cell cancers | 0 | MA, PCR, DNA seq | None | | Schutte et al (1996) |
| Melanoma | 0 | MA, PCR, DNA seq | None | | Schutte et al (1996) |
| Osteosarcoma | 0 | MA, PCR, DNA seq | None | | Schutte et al (1996) |
| | 0 | PCR, SSCP, SB | | | Verbeek et al (1996) |
| Pancreatic cancer | 48 | MA, PCR, DNA seq | Fifteen homozygous deletions, nine intragenic alterations | | Schutte et al (1996) |
| | 53 | PCR, DNA seq | G–A transitions and, more frequently, transversions | Thirty-eight tumours analysed in a three tumour-suppressor gene study | Rozenblum et al (1997) |
| | 27 | LOH, IVSP | Twenty-five homozygous deletions (out of 84), one truncated protein , six point mutations | The original DPC4 study | Hahn et al (1996) |
| Leukaemia | 0 | PCR, SSCP, SB | | | Verbeek et al (1996) |
| Oral cancer | 6 | PCR, SSCP, LOH | Two point mutations, one missense and one substitution | DPC4/DCC study | Watanabe et al (1997) |

SSCP, single strand conformation polymorphism; MA, microsatellite analysis; SB, Southern blot; PCR, polymerase chain reaction; DNA seq, DNA sequencing; LOH, loss of heterozygosity; IVSP, in vitro synthesized protein assay; HNSCC, head and neck squamous cell carcinoma; RT-PCR, reverse transcriptase polymerase chain reaction.

© Cancer Research Campaign 1998

this is in colorectal cancer, in which there is good evidence for low-frequency loss at this locus. Once again, mutations have been identified within the Smad4 gene, strongly arguing against the possibility that Smad4 loss is occurring as a consequence of other genetic events. Using similar evidence, a role for Smad4 can be argued in both lung and oral cancer, although the level of loss in these tumour types is very low. Of the other tumour types listed in Table 1, there is little evidence to directly implicate Smad4, although these studies do provide some support for an involvement of deletions of chromosome 18. The 12% losses of Smad4 in ovarian and breast cancer are perhaps misleading as this estimate of loss is derived from analysis of two cell lines, SW626 and MDA-MB468 respectively.

The relatively restricted tissue specificity of Smad4 inactivation in malignancy had suggested that the other MAD homologues may be targets of tumour suppression in specific tumour subsets. However, analysis of Smads 1, 3, 5 and 6 has revealed no mutations in a total of 167 tumours, including colon, lung, breast and pancreas (Riggens et al. 1997). Recently, 30 human-expressed sequence tags with homology to *Mad*, the *sma* genes of *C. elegans* and/or *Smad4* have been identified and five new genes termed *JV18-1*, *JV15-1*, *JV15-2*, *J5-1* and *JV-4* have been subsequently characterized (Riggens et al. 1996). One gene, *JV18-1*, was localized to 18q21, adjacent to *Smad4* and *DCC*, an area of frequent genetic loss in colorectal carcinoma. *JV18-1* is somatically mutated in 2 out of 18 colorectal carcinomas that had been selected on the basis of loss of heterozygosity of polymorphic markers on 18q (Riggens et al. 1996). Based on homology to other Smad genes, JV18-1 has now been assigned the new nomenclature of Smad2 and is the second *Mad* homologue to be implicated in tumour suppression. The prevalence of *JV18-1/Smad2* mutations in other neoplasms is not well characterized to date, although there is evidence to suggest that Smad2 may be mutated in a subset of leukaemias and lymphomas (Ikezoe et al. 1998) and also in lung carcinomas (approximately 4%) (Uchida et al. 1996). Disruption of the Smad2 gene during development results in a complete loss of embryonic germ-layer tissues (Waldrip et al. 1998), confirming that Smad2, like Smad4, has an essential role to play in development.

TGF-β is well known for its antiproliferative activity in the majority of mammalian epithelial cells, and loss of TGF-β responsiveness is documented to be associated with aggressive neoplasms (Pommier, 1992; Arteaga et al. 1993; Polyak, 1996). It has, therefore, been suggested that loss of components of the TGF-β pathway or its related genes, such as Smad4, would be selected for in the clonal evolution of neoplasms. Significantly, two recent reports (Grau et al. 1997; Atfi et al. 1997) have linked Smad4 to other pathways including the SAPK/JNK cascade, implicating Smad4 in both the control of cell cycle arrest and apoptosis. This suggests that Smad4-dependent malignancy may arise after disruption of these key regulatory mechanisms.

In summary, Smad4 has been shown to be a critical effector of the TGF-β response, a role apparently mediated through its control of other Smad genes. Smad4 appears to be the key regulatory protein of this signalling pathway, ultimately controlling transcription driven by the TGF-β superfamily. Loss of Smad4 has been shown to be associated primarily with pancreatic malignancy, and to a lesser extent with colorectal cancer. Its involvement in other cancer types is currently either very limited or unproven. Characterization of the role played by Smad4 will throw light on the basic biology of pancreatic neoplasia and should also suggest new therapeutic approaches to this disease. Finally, a determination of the role played by Smad4 in malignancy should provide an excellent paradigm for other components of this signalling pathway, perhaps leading to the identification of a family of genes with related TSG activity.

### REFERENCES

Armstrong JF, Kaufman MH, Harrison DJ and Clarke AR (1995) High frequency developmental abnormalities in p53-deficient mice. *Curr Biol* **5**: 937–943

Arteaga CL, Dugger TC, Winnier AR and Forbes JT (1993) Evidence for a positive role of transforming growth factor-beta in human breast cancer cell tumorigenesis. *J Cell Biochem* **7**: 87–193

Atfi A, Buisine M, Mazars A and Gespach C (1997) Induction of apoptosis by DPC4, a transcriptional factor regulated by transforming growth factor-β through stress-activated protein kinase/c-Jun N-terminal kinase (SAPK/JNK) signalling pathway. *J Biol Chem* **272**: 24731–24734

Attisano L, Carcamo J, Ventura F, Weis FMB, Massague J and Wrana JL (1993) Identification of human activin and TGF-beta type-I receptors that form heteromeric kinase complexes with type-II receptors. *Cell* **75**: 671–680

Barrett MT, Schutte M, Kern SE and Reid BJ (1996) Allelic loss and mutational analysis of the DPC4 gene in esophageal adenocarcinoma. *Cancer Res* **56**: 4351–4353

Caestecker MP de, Hemmati P, Larisch-Bloch S, Ajmera R, Roberts AB and Lechleider RJ (1997) Characterisation of functional domains within Smad4/DPC4. *J Biol Chem* **272**: 13690–13696

Chen X, Weisberg E, Fridmacher V, Watanabe M, Naco G and Whitman M (1997) Smad4 and FAST-1 in the assembly of activin-responsive factor. *Nature* **389**: 85–89

Clarke AR, Maandag ER, Vanroon M, Vanderlugt NMT, Vandervalk M, Hooper ML, Berns A and Riele HT (1992) Requirement for a functional Rb-1 gene in murine development. *Nature* **359**: 328–330

Derynck R, Gelbart WM, Harland RM, Heldin CH, Kern SE, Massague J, Melton DA, Mlodzik MB, Padgett RW, Roberts AB, Smith J, Thomsen H, Vogelstein B and Wang XF (1996) Nomenclature – vertebrate mediators of TGF-beta family signals. *Cell* **87**: 173

Grau AM, Zhang L, Wang WX, Ruan SB, Evans DB, Abbruzzese JL, Zhang W and Chiao PJ (1997) Induction of p21 (Waf1) expression and growth inhibition by transforming growth factor beta involve the tumor suppressor gene DPC4 in human pancreatic adenocarcinoma cells. *Cancer* **57**: 3929–3934

Hahn SA, Schutte M, Hoque ATMS, Moskaluk CA, Dacosta LT, Rozenblum E, Weinstein CL, Fischer A, Yeo CJ, Hruban RH and Kern SE (1996*a*) DPC4, a candidate tumor-suppressor gene at human chromosome 18q21.1. *Science* **271**: 350–353

Hahn SA, Hoque ATMS, Moskaluk CA, Dacosta LT, Schutte M, Rozenblum E, Seymour AB, Weinstein CL, Yeo CJ, Hruban RH and Kern SE (1996*b*) Homozygous deletion map at 18q21.1 in pancreatic cancer. *Cancer Res* **56**: 490–494

Heldin CH, Miyazono K and ten Dijke P (1997) TGF-beta signalling from cell membrane to nucleus through SMAD proteins. *Nature* **390**: 465–471

Hoque ATMS, Hahn SA, Schutte M and Kern SE (1997) DPC4 gene mutation in colitis associated neoplasia. *Gut* **40**: 120–122

Ikezoe T, Takeuchi S, Kamioka M, Daibata M, Kubonishi I, Taguchi H and Miyoshi I (1998) Analysis of the Smad2 gene in haematological malignancies. *Leukemia* **12**: 94–95

Kim SK, Fan YH, Papadimitrakopoulou V, Clayman G, Hittelman WN, Hong WK, Lotan R and Mao L (1996) DPC4, a candidate tumor-suppressor gene, is altered infrequently in head and neck squamous-cell carcinoma. *Cancer Res* **56**: 2519–2521

Kingsley DM (1994) The TGF-beta superfamily – new members, new receptors, and new genetic tests of function in different organisms. *Genes Dev* **8**: 133–146

Kong XT, Choi SH, Inoue A, Takita J, Yokota J, Kanada R, Yamamoto K, Bessho F, Yanagisawa M and Hayashi Y (1997) DPC4 gene mutation in colitis associated neoplasia. *Eur J Cancer* **33**: 1962–1965

Knudson AG (1971) A statistical study of retinoblastoma. *Proc Natl Acad Sci USA* **68**: 820–823

Lagna G, Hata A, Hemmatibrivanlou A and Massague J (1996) Partnership between DPC4 and Smad proteins in TGF-beta signaling pathways. *Nature* **383**: 832–836

Lei JY, Zou TT, Shi YQ, Zhou XI, Smolinski KN, Yin J, Souza RF, Appel R, Wang S, Cymes K, Chan O, Abraham JM, Harpaz N and Meltzer SJ (1996) Infrequent DPC-1 gene mutation in esophageal cancer, gastric cancer and ulcerative colitis-associated neoplasms. *Oncogene* **13**: 2459–2462

© Cancer Research Campaign 1998

Liu F, Hata A, Baker JC, Doody J, Carcamo J, Harland RM and Massague J (1996) Human MAD protein acting as a BMP-regulated transcriptional activator. *Nature* **381**: 620–623

Liu F, Pouponnot C and Massague J (1997) Dual role of the Smad4/DPC4 tumor suppressor in TGF beta-inducible transcriptional complexes. *Genes Dev* **1**: 3157–3167

Macgrogan D, Pegram M, Slamon D and Bookstein R (1997) Comparative mutational analysis of DPC4 (Smad4) in prostatic and colorectal carcinomas. *Oncogene* **15**: 1111–1114

Maciassilva M, Abdollah S, Hoodless PA, Pirone R, Attisano L and Wrana JL (1996) MADR2 is a substrate of the TGF beta receptor and its phosphorylation is required for nuclear accumulation and signaling. *Cell* **86**: 1215–1224

Massague J, Attisano L, Carcamo J, Lopezcasillas F, Doody J, Wrana JL and Zentella A (1993) TGF-beta signals growth inhibition through a heteromeric kinase receptor. *J Cell Biochem* **17**: 185

Moser AR, Pitot HC and Dove WF (1990) A dominant mutation that predisposes to multiple intestinal neoplasia in the mouse. *Science* **247**: 322–324

Nagatake M, Takagi Y, Osada N, Uchida K, Mitsudomi T, Saji S, Shimokata K, Takahashi T and Takahashi T (1996) Somatic in vivo alterations of the DPC4 gene at 18q21 in human lung cancers. *Cancer Res* **56**: 2718–2720

Piao Z, Kim H, Jeon BK, Lee WJ and Park C (1997) Relationship between loss of heterozygosity of tumor suppressor genes and histologic differentiation in hepatocellular carcinoma. *Cancer* **80**: 865–872

Polyak K (1996) Negative regulation of cell growth by TGF-beta. *Biochim Biophys Acta – Rev Cancer* **1242**: 185–199

Pommier G (1992) Growth factors and intestinal neoplasms. *Bull Cancer* **79**: 427–449

Powell SM, Harper JC, Hamilton SR, Robinson CR and Cummings OW (1997) Inactivation of Smad4 in gastric carcinomas. *Cancer Res* **57**: 4221–4224

Riggins GJ, Thiagalingam S, Rozenblum E, Weinstein CL, Kern SE, Hamilton SR, Willson JKY, Markowitz SD, Kinzler KW and Vogelstein B (1996) MAD-related genes in the human. *Nature Genet* **13**: 347–349

Riggins GJ, Kinzler KW, Vogelstein B and Thiagalingam S (1997) Frequency of Smad gene mutations in human cancers. *Cancer Res* **57**: 2578–2580

Rozenblum E, Schutte M, Goggins M, Hahn SA, Panzer S, Zahurak M, Goodman SN, Sohn TA, Hruban RH, Yeo CJ and Kern SE (1997) Tumor-suppressive pathways in pancreatic carcinoma. *Cancer Res* **57**: 1731–1734

Sah VP, Atardi LD, Mulligan DJ, Williams BO, Bronson RT and Jacks T (1995) A subset of p53-deficient embryos exhibit exencephaly. *Nature Genet* **109**: 175–180

Savage C, Das P, Finelli AL, Townsend SR, Sun CY, Baird SE and Padgett RW (1996) *Caenorhabditis elegans* genes *Sma2*, *Sma-3*, and *Sma-4* define a conserved family of transforming growth factor-beta pathway components. *Proc Natl Acad Sci USA* **93**: 790–794

Schutte M, Hruban RH, Hedrick L, Cho KR, Nadasdy GM, Weinstein CL, Bova GS, Isaacs WB, Cairns P, Nawroz H, Sidransky D, Casero RA, Meltzer PS, Hahn SA and Kern SE (1996) DPC4 gene in various tumor types. *Cancer Res* **56**: 2527–2530

Sekelsky JJ, Newfeld SJ, Raftery LA, Chartoff EH and Gelbart WM (1995) Genetic characterization and cloning of mothers against dpp, a gene required for decapentaplegic function in *Drosophila melanogaster*. *Genetics* **139**: 1347–1358

Shi YG, Hata A, Lo RS, Massague J and Pavletich NP (1997) A structural basis for mutational inactivation of the tumour suppressor Smad4. *Nature* **388**: 87–93

Sirard C, Delapompa JL, Elia A, Itie A, Mirtsos C, Cheung A, Hahn S, Wakeham A, Schwartz L, Kern SE, Rossant J and Mak TW (1998) The tumor suppressor gene Smad4/DPC4 is required for gastrulation and later for anterior development of the mouse embryo. *Genes Dev* **12**: 107–119

Takagi Y, Kohmura H, Futamura M, Kida H, Tanemura H, Shimokawa K and Saji S (1996) Somatic alterations of the DPC4 gene in human colorectal cancers in vivo. *Gastroenterology* **111**: 1369–1372

Takaku K, Oshima M, Miyoshi H, Matsui M, Seldin M and Taketo MM (1998) Intestinal tumorigenesis in compound mutant mice of both *Dpc4* (Smad4) and *Apc* genes. *Cell* **92**: 645–656

Thiagalingam S, Lengauer C, Leach FS, Schutte M, Hahn SA, Overhauser J, Willson JKV, Markowitz S, Hamilton SR, Kern SE, Kinzler KW and Vogelstein B (1996) Evaluation of candidate tumor-suppressor genes on chromosome 18 in colorectal cancers. *Nature Genet* **13**: 343–346

Uchida K, Nagatake M, Osada H, Yatabe Y, Kondo M, Mitsudomi T, Masuda A, Takahashi T and Takahashi T (1996) Somatic in vivo alterations of the Jv18-1 gene at 18q21 in human lung cancers. *Cancer Res* **56**: 5583–5585

Verbeek W, Spirin K, Hatta Y, Miller C, Kawamata N, Takeuchi S, Koike M, Asou H, Simpson JF and Koeffler HP (1997) DPC4/Smad4 in non-pancreatic tumors with frequent LOH 18q21 and in hematological malignancies. *Int J Oncol* **10**: 257–260

Waldrip WR, Bikoff EK, Hoodless PA, Wrana JL and Robertson EJ (1998) Smad2 signaling in extraembryonic tissues determines anterior–posterior polarity of the early mouse embryo. *Cell* **92**: 797–808

Watanabe T, Wang XL, Miyakawa A, Shiiba M, Imai Y, Sato T and Tanzawa H (1997) Mutational state of tumor suppressor genes (DCC, DPC4) and alteration on chromosome 18q21 in human oral cancer. *Int J Oncol* **11**: 1287–1290

Wrana JL and Attisano L (1996) MAD-related proteins in TGF-beta signalling. *Trends Genet* **12**: 493–496

Wrana JL, Carcamo J, Attisano L, Cheifetz S, Zentella A, Lopezcasillas F and Massague J (1992*a*) The type-II TGF-beta receptor signals diverse responses in cooperation with the type-I receptor. *CSH Symp Quant Biol* **57**: 81–86

Wrana JL, Attisano L, Carcamo J, Zentella A, Doody J, Laiho M, Wang XF and Massague J (1992*b*) TGF-beta signals through a heteromeric protein-kinase receptor complex. *Cell* **71**: 1003–1014

Xin C, Rubock MJ and Whitman M (1996) A transcriptional partner for MAD proteins in TGF-beta signaling (vol. **383**: 691, 1996). *Nature* **348**: 648

© Cancer Research Campaign 1998